# Regulation of Sox9 activity by crosstalk with nuclear factor-κB and retinoic acid receptors

**DOI:** 10.1186/ar2349

**Published:** 2008-01-09

**Authors:** Jason S Rockel, Julie C Kudirka, Andrew J Guzi, Suzanne M Bernier

**Affiliations:** 1 Canadian Institutes of Health Research Group in Skeletal Development and Remodeling, and Department of Anatomy and Cell Biology, Schulich School of Medicine & Dentistry, The University of Western Ontario, London, Ontario, Canada, N6A 5C1

## Abstract

**Introduction:**

Sox9 and p300 cooperate to induce expression of cartilage-specific matrix proteins, including type II collagen, aggrecan and link protein. Tumour necrosis factor (TNF)-α, found in arthritic joints, activates nuclear factor-κB (NF-κB), whereas retinoic acid receptors (RARs) are activated by retinoid agonists, including all-trans retinoic acid (atRA). Like Sox9, the activity of NF-κB and RARs depends upon their association with p300. Separately, both TNF-α and atRA suppress cartilage matrix gene expression. We investigated how TNF-α and atRA alter the expression of cartilage matrix genes.

**Methods:**

Primary cultures of rat chondrocytes were treated with TNF-α and/or atRA for 24 hours. Levels of transcripts encoding cartilage matrix proteins were determined by Northern blot analyses and quantitative real-time PCR. Nuclear protein levels, DNA binding and functional activity of transcription factors were assessed by immunoblotting, electrophoretic mobility shift assays and reporter assays, respectively.

**Results:**

Together, TNF-α and atRA diminished transcript levels of cartilage matrix proteins and Sox9 activity more than each factor alone. However, neither agent altered nuclear levels of Sox9, and TNF-α did not affect protein binding to the *Col2a1 *48-base-pair minimal enhancer sequence. The effect of TNF-α, but not that of atRA, on Sox9 activity was dependent on NF-κB activation. Furthermore, atRA reduced NF-κB activity and DNA binding. To address the role of p300, we over-expressed constitutively active mitogen-activated protein kinase kinase kinase (caMEKK)1 to increase p300 acetylase activity. caMEKK1 enhanced basal NF-κB activity and atRA-induced RAR activity. Over-expression of caMEKK1 also enhanced basal Sox9 activity and suppressed the inhibitory effects of TNF-α and atRA on Sox9 function. In addition, over-expression of p300 restored Sox9 activity suppressed by TNF-α and atRA to normal levels.

**Conclusion:**

NF-κB and RARs converge to reduce Sox9 activity and cartilage matrix gene expression, probably by limiting the availability of p300. This process may be critical for the loss of cartilage matrix synthesis in inflammatory joint diseases. Therefore, agents that increase p300 levels or activity in chondrocytes may be useful therapeutically.

## Introduction

Members of the Sry-type high mobility group box (Sox) transcription factor family are regulators of tissue-specific gene expression (for review, see Wegner [1]). A subset of Sox proteins is responsible for controlling cartilage development and chondrocyte function by regulating the expression of specific matrix genes. L-Sox5, Sox6 and Sox9 are necessary regulators for induction and maintenance of cartilage-specific collagen expression by chondrocytes [2]. L-Sox5 and Sox6 heterodimerize via inherent leucine zippers and bind DNA, but they lack transactivation domains necessary to control transcription. In contrast, Sox9 contains a carboxyl-terminal transactivation domain and probably serves to regulate gene transcription directly. Consequently, L-Sox5 and Sox6 synergize with Sox9 to induce transcription of the type II collagen gene by binding to the 48-base-pair (bp) minimal enhancer region [2-4]. Sox9 also coordinates expression of two other cartilage extracellular matrix molecules, aggrecan and link protein, through activity at regulatory regions of each gene [5,6].

Homeostatic maintenance of mature cartilage is characterized by continual production and degradation of extracellular matrix, processes that are coordinated in chondrocytes via factors such as the Sox proteins. In contrast, cartilage degeneration in joint diseases such as rheumatoid arthritis and osteoarthritis results from a shift in this balance toward catabolism. One factor found in the synovial fluid of rheumatoid and osteoarthritic patients is the inflammatory cytokine tumour necrosis factor (TNF)-α [7-9]. In addition to upregulating the expression of catabolic factors such as matrix metalloproteinases and aggrecanases [10,11], TNF-α downregulates expression of transcripts for type II collagen, aggrecan and link protein by chondrocytes [12-14]. Signalling events that mediate these changes in gene expression include activation of nuclear factor-κB (NF-κB), extracellular signal-regulated kinases 1/2 and p38 mitogen-activated protein kinase pathways, and decreases in Sox9 protein expression [13-15].

Inhibitor of NF-κB (IκB) mask the nuclear localization signal on NF-κB. When cells are stimulated by TNF-α, IκB is phosphorylated by activated IκB kinase. Phosphorylated IκB is ubiquitinated and degraded by the 26S proteasome, permitting NF-κB isoforms to homodimerize or heterodimerize, enter the nucleus and alter gene transcription (for review, see Karin and Ben-Neriah [16]). Preventing the activation of NF-κB allows Sox9 to retain its full activity at the type II collagen enhancer in the presence of TNF-α [13], suggesting a relationship between these transcription factors.

Some transcription factors can be directly regulated by binding of membrane-permeable agonists. An example of such an agonist is all-trans retinoic acid (atRA), a metabolite of vitamin A, that is bound preferentially by the α, β and γ retinoic acid receptors (RARs) [17]. RARs act as homodimers or heterodimers with members of the retinoid X receptor family to transactivate genes.

Regulation of retinoid signalling is critical for the development and maintenance of cartilage. In chondroprogenitor cells, activation of retinoid receptors, particularly RARα, decreases Sox9 activity at the type II collagen enhancer and reduces the content of glycosaminoglycans in the extracellular matrix. Moreover, treatment of equine articular cartilage explants with atRA results in loss of glycosaminoglycans as a consequence of reduced proteoglycan synthesis [18]. Furthermore, mice fed a diet high in retinyl acetate, a synthetic derivative of vitamin A, exhibit cartilage atrophy and other osteoarthritic characteristics [19]. In addition, increasing retinoid levels in humans accelerates hypertrophy of chondrocytes and ossification (hyperostosis), which contributes to the progression of degenerative joint diseases [20]. Thus, retinoids, including atRA, appear to have negative effects on matrix synthesis and homeostasis in cartilage.

Interestingly, adult mice fed diets deficient in vitamin A exhibit generalized increases in basal NF-κB activity [21]. Upon administration of retinoic acid, NF-κB activity is reduced, which suggests an effect of RARs on function rather than expression of NF-κB. Recombinant NF-κB p65 and members of both the RAR and retinoid X receptor families of retinoic acid receptors associate with each other *in vitro *[22]. Furthermore, addition of an atRA analogue to macrophage cell extracts decreases TNF-α-induced binding of NF-κB to DNA [22]. Consistent with these results, administration of atRA reduces joint destruction in collagen-induced inflammatory arthritis [23].

Co-factors associated with regulatory transcription factors play vital roles in the transactivation of genes. Sox9, NF-κB and RARs share the common co-factor p300, an acetylase that is required for full activity of these transcription factors [24-26]. In the present study, we investigated the mechanism by which such transcription factors integrate to regulate gene expression. We determined the effect of co-activation of NF-κB and RARs on Sox9 function and cartilage matrix gene expression. We found that atRA and TNF-α signalling converge in the nucleus to promote greater reductions in Sox9 activity and matrix gene expression than signalling from each alone. These reductions in Sox9 activity are consistent with limitation in the availability of p300 for Sox9 when NF-κB and RARs are also activated. Thus, agents that promote p300 activity or availability may be useful therapeutically to maintain cartilage matrix production in inflammatory joint disease.

## Materials and methods

### Cell culture

Primary chondrocytes were isolated (8 to 12 × 10^5 ^cells/rat) from the femoral condyles of 1-day-old Sprague Dawley rats (Charles River, St. Hyacinthe, Quebec, Canada), as described previously [13]. The Animal Use Subcommittee of the University of Western Ontario Council on Animal Care approved the use of rats in these studies. Chondrocytes were plated on tissue culture plastic (Falcon, Franklin Lakes, NJ, USA) at a density of 3.0 to 4.25 × 10^4 ^cells/cm^2 ^and grown in RPMI-1640 media supplemented with 5% foetal bovine serum, 100 U/ml penicillin, 100 μg/ml streptomycin and 10 mmol/l HEPES (Invitrogen Life Technologies Inc., Burlington, Ontario, Canada). The medium was changed every 3 days until cultures reached confluence, typically after 6 to 8 days.

### RNA isolation, Northern blot hybridization and real-time PCR

Total RNA was isolated using Trizol (Invitrogen Life Technologies Inc.) and quantified spectrophotometrically. Northern blot hybridizations were performed as described previously [13] using probes corresponding to the C-propeptide of mouse type II collagen (pKN225) [3], rat aggrecan core protein (p1353) [27] and mouse 18S rRNA (Ambion, Austin, TX, USA), radiolabelled with [α^32^P]-dCTP (3000 Ci/mmol; NEN, Boston, MA, USA) using a random-primed oligonucleotide method (Prime-a-gene labeling kit; Promega, Madison, WI, USA). For quantitative real-time PCR (qPCR) analysis, total RNA was processed using an RNeasy Mini Kit (Qiagen, Mississauga, Ontario, Canada). Amplification reactions were prepared by adding RNA (25 ng) to TaqMan One Step RT-PCR Master Mix (4309169; Applied Biosystems Inc., Streetsville, Ontario, Canada) containing primers to rat type II collagen (Rn00564954_m1), aggrecan 1 (Rn00573424_m1), link protein (Rn00569884_m1), or GAPDH (glyceraldehyde-3-phosphate dehydrogenase; 4308313; Applied Biosystems Inc.). Reverse transcription and qPCR reactions were performed in a Prism 7900 HT Sequence Detector (Applied Biosystems Inc.). Briefly, samples were incubated for 30 min at 48°C to make cDNA templates, followed by a maximum of 40 amplification cycles, alternating between 95°C for 15 seconds and 60°C for 1 minute. Results were analyzed using SDS v2.1 software (Applied Biosystems Inc.).

### Luciferase reporter analysis

Cells from confluent cultures were detached using trypsin-EDTA (Invitrogen Life Technologies Inc.), pelleted, resuspended in serum-free culture medium, plated into 48-well dishes (3.4 × 10^4 ^cells/well) and transfected with reporter plasmids. The reporter plasmids included the following: a κB reporter (BD Biosciences, Mississauga, Ontario, Canada), comprising four tandem repeats of the κB response element upstream of the firefly luciferase reporter sequence, expressed upon NF-κB activation; a retinoic acid response element (RARE) firefly luciferase reporter (pWl-β RARE_3_-luc), regulated by activation of RARs via retinoic acid [28]; and a type II collagen enhancer luciferase reporter containing four repeats of the 48-bp minimal enhancer of the type II collagen gene (pGL3 [4 × 48]) [28], each with a binding site for Sox9. Previous studies have shown that multiple repeats of the minimal enhancer are required for optimal output [29]. For some experiments, chondrocytes were co-transfected with reporter constructs and expression constructs for constitutively active mitogen-activated protein kinase kinase kinase (caMEKK)1 (Clontech, Mountain View, CA, USA), a phosphorylation site-deficient IκB (IκB-2N; pSVK3-IκB-2NΔ4) [30], or p300. In all experiments, chondrocytes were co-transfected with a 0.002 μg renilla luciferase plasmid (pRL-SV40; Promega), which was used in most cases to control for transfection efficiency.

Suspended chondrocytes were transfected with equal amounts of DNA (0.052 μg of each vector and additional pBluescript vector plasmid [Stratagene, La Jolla, CA, USA] if necessary), using 2.25 μl Fugene 6 transfection reagent (Roche Diagnostics Corporation, Indianapolis, IN, USA) per microgram of DNA. After 24 hours of incubation in serum-containing culture medium, chondrocytes were incubated in serum-free medium (control medium), TNF-α (Sigma Aldrich, Mississauga, Ontario, Canada), atRA (Sigma Aldrich), or a combination of TNF-α and atRA for 24 hours. Luciferase activity was measured using the Dual Luciferase Assay System (Promega) in an L-max II microplate reader (Molecular Devices, Sunnyvale, CA, USA).

In experiments involving over-expression of p300, we noted a mean (± standard deviation) fold increase in activity of 1.6 ± 0.2 (*P *< 0.03) of the SV40 constitutive promoter, which drives the expression of renilla luciferase. Therefore, in these experiments, only Sox9-driven luciferase activity was analyzed.

### Antibodies

Antibodies used in these studies were anti-Sox9 (H-90), anti-NF-κB p65 (C-20) and anti-RARα (C-20) from Santa Cruz Biotechnology (Santa Cruz, CA, USA); anti-β-catenin (C-2206) from Sigma Aldrich; and horseradish peroxidase-conjugated goat-anti-rabbit secondary antibody from Pierce Biotechnology Inc. (Rockford, IL, USA).

### Preparation of nuclear extracts, immunoblotting, and electrophoretic mobility shift assays

Confluent cultures were serum-deprived overnight before addition of TNF-α and/or atRA for 24 hours. Nuclear extracts were prepared using a method modified from that reported by Dignam and coworkers [31], as previously described [13]. Equal concentrations (30 μg) of nuclear protein extracts were resolved by electrophoresis on 7.5% SDS-polyacrylamide gels. Proteins were transferred onto nitrocellulose membrane (Protran; Schleicher & Schuell, Keene, NH, USA) by electroblotting, and equivalency of loading was verified by staining with Ponceau red. Membranes were blocked in 5% nonfat milk (Carnation, North York, Ontario, Canada) in Tris-buffered saline plus 0.05% Tween 20 for 1 hour, followed by incubation with the primary antibody overnight in blocking buffer. Membranes were washed with Tris-buffered saline plus 0.05% Tween 20 and incubated with horseradish peroxidase-conjugated secondary antibody. Protein-antibody complexes were visualized using SuperSignal West Pico Chemiluminescent Substrate (Pierce Biotechnology Inc.), followed by exposure to Hyperfilm-ECL (Amersham Biosciences, Baie D'Urfé, Quebec, Canada). Membranes were stripped using 1 mol/l glycine pH 2.5 before re-probing. For NF-κB p65 and RARα immunoblots, relative band intensities were calculated as the ratio of the band intensity of NF-κB p65 or RARα to that of β-catenin that served as a loading control.

Binding of nuclear protein complexes to the κB response element or the *Col2a1 *48-bp minimal enhancer sequence was determined by electrophoretic mobility shift assays (EMSAs), as described previously [13]. The double-stranded oligonucleotide containing the κB cognate sequence (5'-AGTTGAGGGGACTTTCCCAGG-3') was purchased from Santa Cruz Biotechnology. The double-stranded oligonucleotide containing the rat *Col2a1 *48-bp minimal enhancer (5'-CTGTGAATCGGGCTCTGTATGCACTCGAGAAAAGCCCCATTCATGAGA-3'), described by Lefebvre and coworkers [29], was obtained from Invitrogen Life Technology. Supershift or antibody interference assays were performed by adding antibodies against NF-κB (2 μg) or RARα (2 μg) to the nuclear extract/DNA complex reaction for 1 hour before electrophoresis on 4% polyacrylamide gels. Following electrophoresis, gels were dried and exposed to Hyperfilm-MP (Amersham Biosciences) at -80°C.

### Densitometry and statistical analyses

Immunoblot films were analyzed by densitometry using Kodak Digital Science software (Eastman Kodak, Rochester, NY, USA). Data were analyzed by paired *t*-tests, or by analysis of variance followed by Tukey's multiple comparisons tests. Normalized data were log or arcsine transformed before analysis (PRISM v2.0 software; GraphPad Software Inc., San Diego, CA, USA). Unlabelled bars or bars labelled with the same lower case letters are not significantly different from each other (*P *> 0.05).

## Results

### Co-treatment with TNF-α and atRA further reduces expression of extracellular matrix protein genes

**Figure 1:**
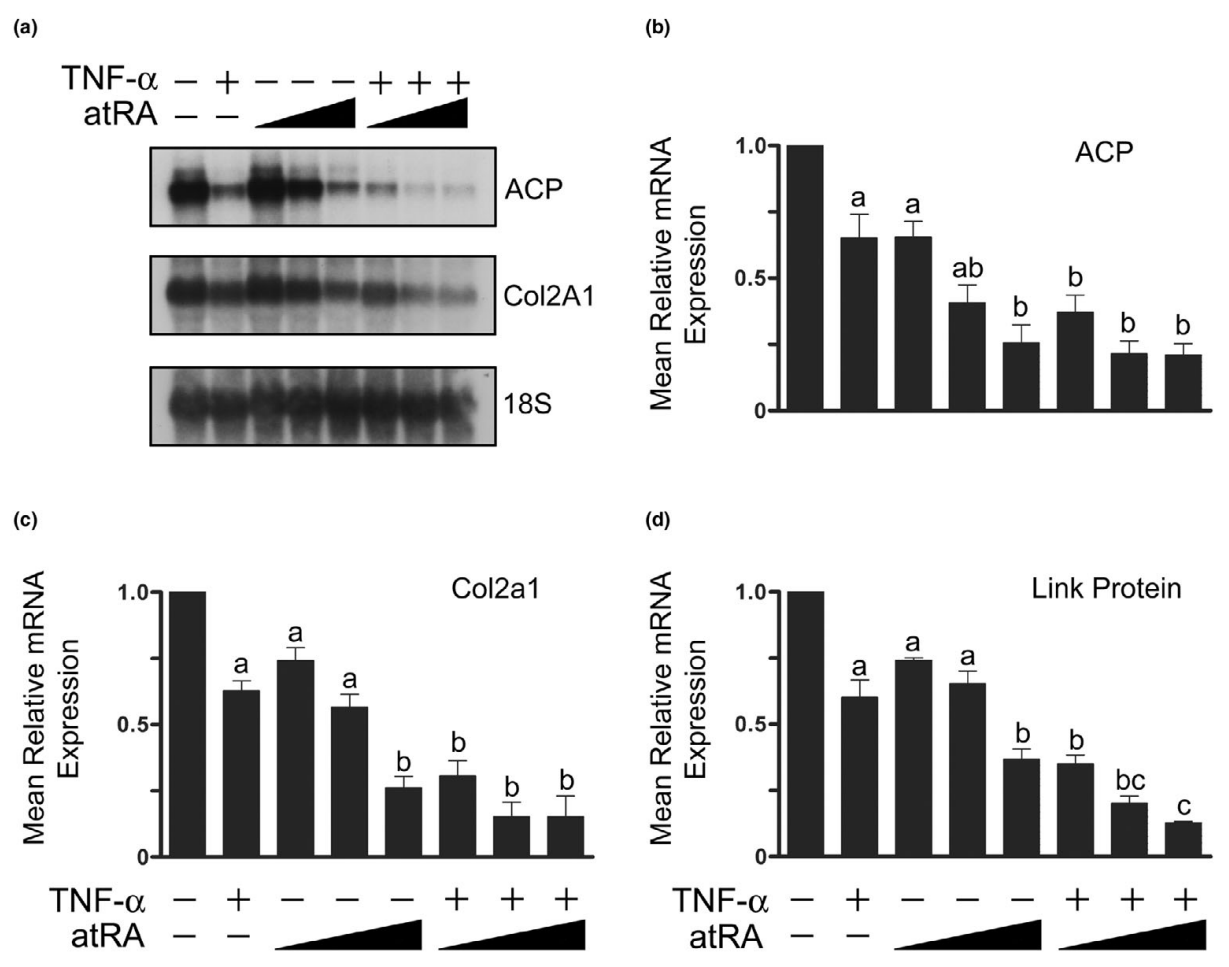
Effects of TNF-α and atRA on matrix gene expression. Chondrocytes were treated with or without tumour necrosis factor (TNF)-α (30 ng/ml) and/or all-trans retinoic acid (atRA; 1, 10, or 100 nmol/l) for 24 hours. Total RNA was evaluated for aggrecan core protein (ACP), type II collagen (Col2a1) and 18S rRNA by **(a) **Northern blot analysis and by **(b, c) **quantitative real-time PCR, whereas link protein mRNA was evaluated only by **(d) **quantitative real-time PCR. TNF-α and atRA alone decreased aggrecan, type II collagen and link protein transcript levels. Co-treatment with TNF-α and atRA further decreased mRNA transcript levels of aggrecan, type II collagen and link protein. Panel a: Northern blots are representative of three independent experiments; 18S rRNA levels were used to verify equal loading. Panels b to d: data are ratios of matrix gene: *Gapdh *transcript levels normalized as a fraction of the ratios in untreated cultures (first bar), and are expressed as means ± standard error (the number of independent experiments was seven for panel b, five for panel c, and five for panel d). Data were evaluated by one-way analysis of variance and Tukey's multiple comparisons test. Unlabelled bars or bars labelled with the same lower case letters are not significantly different (*P *> 0.05).

We first investigated how expression of extracellular matrix genes responded to TNF-α and atRA. Chondrocytes were treated for 24 hours with TNF-α and/or increasing concentrations of atRA. Following treatment, transcript levels of type II collagen, aggrecan core protein and link protein were determined by Northern blot analysis and/or qPCR (Figure 1). TNF-α significantly reduced levels of type II collagen, aggrecan core protein and link protein mRNA. Treatment of cells with atRA reduced mRNA levels of all three matrix genes in a concentration-dependent manner. Interestingly, co-treatment of cells with TNF-α and atRA decreased levels of these transcripts more than each factor alone. These results suggest that signalling from TNF-α and atRA converge to influence the activity of transcription factors, such as Sox9, that are necessary for the expression of cartilage matrix genes.

### Effects of TNF-α and atRA on Sox9 activity

**Figure 2:**
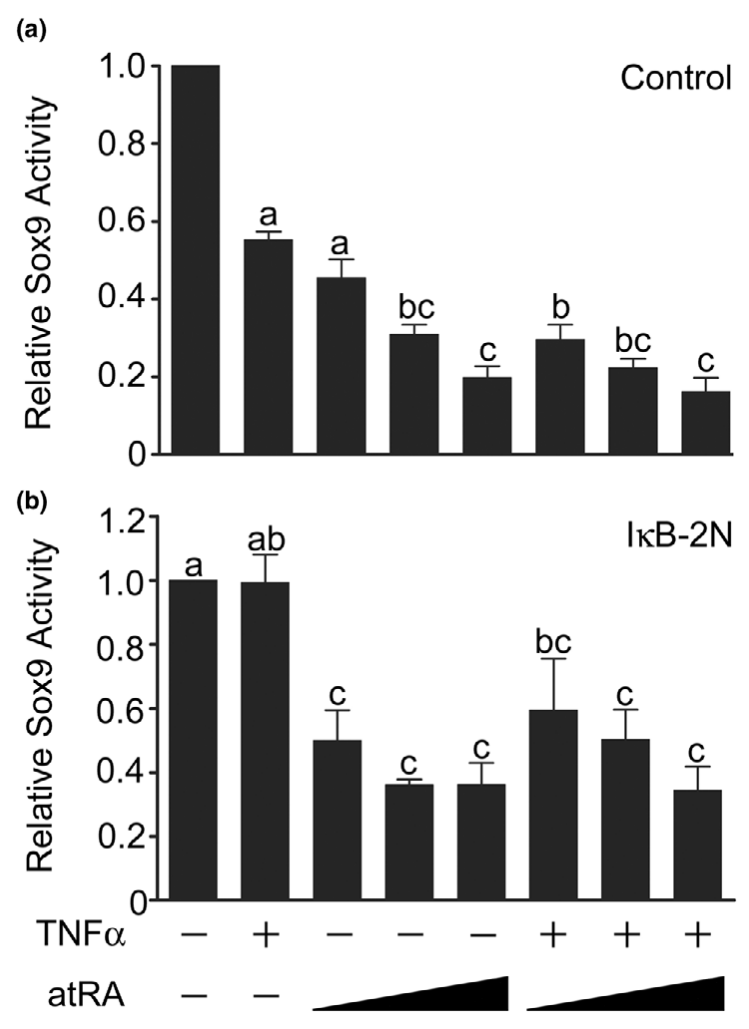
Effects of TNF-α and atRA on Sox9 activity. Chondrocytes were transfected with **(a) **the type II collagen enhancer luciferase reporter and some cultures were also co-transfected with **(b) **a phosphorylation site-deficient inhibitor of nuclear factor-κB (IκB-2N). Cultures were then treated with or without tumour necrosis factor (TNF)-α (30 ng/ml) and/or all-trans retinoic acid (atRA; 1, 10, or 100 nmol/l) for 24 hours. Panels a: TNF-α and atRA separately decreased Sox9 activity. Greater decreases in Sox9 activity were induced by treatment with TNF-α and atRA (1 nmol/l). Panel b: IκB-2N did not significantly increase basal levels of Sox9 activity (1.3 ± 0.2 fold increase [mean ± standard deviation]) compared with untreated cells transfected with Sox9 reporter only (*P *> 0.05). IκB-2N attenuated the effect of TNF-α on Sox9 activity, but regulation of Sox9 activity by atRA was maintained. Data are ratios of Sox9-regulated firefly luciferase units to constitutive SV40-regulated renilla luciferase units, normalized as a fraction of the ratios in untreated cultures (first bar), and are expressed as means ± standard error (the number of independent experiments was six for panel a and four for panel b). Data were evaluated by repeated measures analysis of variance and Tukey's multiple comparisons test. Unlabelled bars or bars labelled with the same lower case letters are not significantly different (*P *> 0.05).

We investigated the activity of Sox9 using a reporter construct based on the type II collagen minimal enhancer (Figure 2a). TNF-α significantly reduced Sox9 reporter activity (approximately 47%). Sox9 reporter activity also decreased with increasing concentrations of atRA. At 10^-9 ^mol/l atRA, co-treatment with TNF-α resulted in a further decrease in Sox9 reporter activity, which is consistent with the observed changes in expression of cartilage matrix protein transcripts.

Previously, we found that regulation of Sox9 activity at the type II collagen enhancer was dependent on NF-κB activation [13]. To determine whether the effects of TNF-α and atRA were both mediated by NF-κB, we restricted NF-κB nuclear translocation by over-expression of IκB-2N – an IκBα that is resistant to phosphorylations required for NF-κB release (Figure 2b). IκB-2N did not significantly alter basal Sox9 activity or the reduction of Sox9 activity following atRA treatment alone. In contrast, IκB-2N did eliminate the further reduction observed in the presence of both TNF-α and atRA (10^-9 ^mol/l). These results indicate that reduction of Sox9 activity by atRA is independent of NF-κB activation.

### Binding of protein complexes to the Col2a1 48-bp minimal enhancer and nuclear Sox9 levels

**Figure 3:**
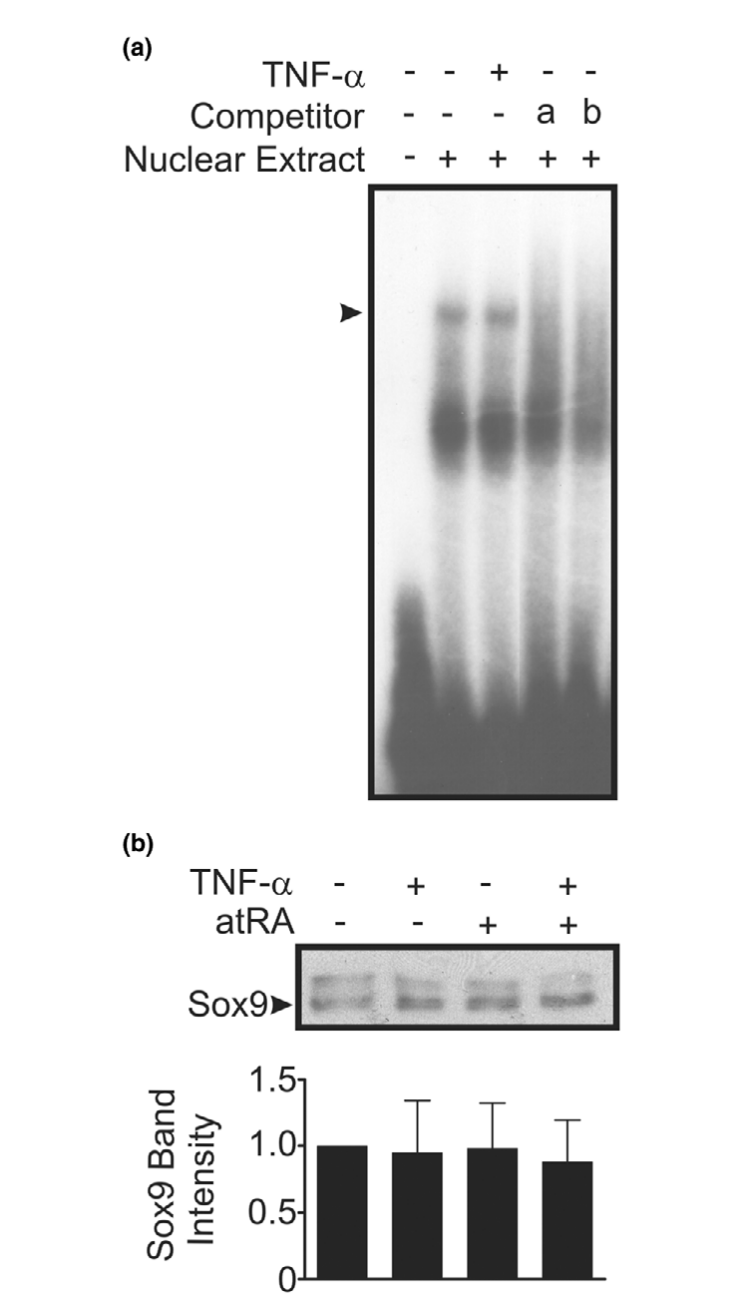
Effect of TNF-α on Sox9-DNA binding and Sox9 nuclear protein levels. **(a) **Chondrocytes were treated for 24 hours with or without tumour necrosis factor (TNF)-α (30 ng/ml). Nuclear extracts (10 μg) were incubated with double-stranded ^32^P-labelled oligonucleotides corresponding to the *Col2a1 *minimal enhancer sequence and resolved on a 4% polyacrylamide gel. Where indicated, excess unlabelled specific oligonucleotides (a: 40× or b: 80×) were added as competitors. TNF-α did not change the amount of protein complex (arrowhead) bound to the oligonucleotide. Results shown are representative of three independent experiments. **(b) **Chondrocytes were treated for 24 hours with or without TNF-α (30 ng/ml) and/or atRA (100 nmol/l). Nuclear extracts (30 μg) were resolved on a 7.5% polyacrylamide gel and immunoblotted with antibody recognizing Sox9. The 64 kDa band corresponding to Sox9 (arrowhead) was quantified by densitometry. Data were normalized as a fraction of band density in untreated chondrocytes and are expressed as means ± standard error (three independent experiments). Data were evaluated by one-way analysis of variance. There was no significant change in the level of Sox9 nuclear protein (*P *> 0.05).

Since Sox9 activity was decreased by TNF-α and atRA, we determined whether there were changes in protein complex binding to the *Col2a1 *48-bp minimal enhancer sequence or alterations in nuclear levels of Sox9. Chondrocytes were treated with TNF-α and nuclear extracts were analyzed by EMSA. TNF-α did not change the amount of protein complex bound to the 48-bp minimal enhancer sequence (Figure 3a). In addition, TNF-α and atRA (alone or in combination) did not change nuclear levels of Sox9 assessed by immunoblot (Figure 3b). Taken together, these findings indicate that the observed changes in Sox9 activity are independent of changes in DNA binding or nuclear protein levels.

### atRA reduces NF-κB activity in a concentration-dependent manner

**Figure 4:**
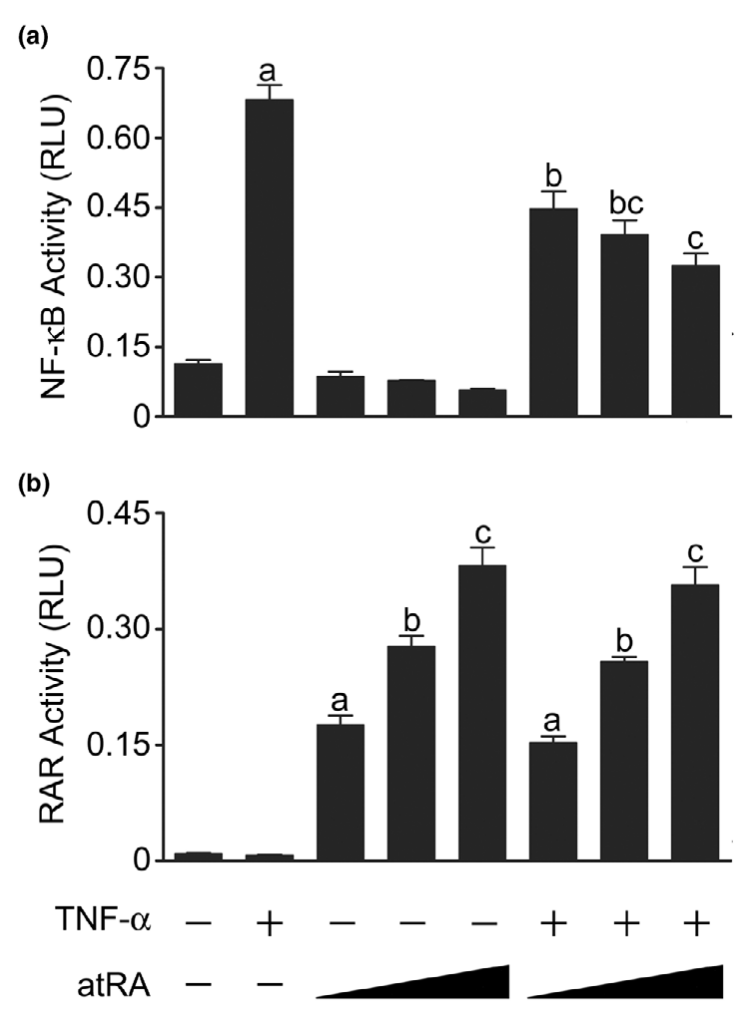
Effect of atRA on NF-κB and RAR activity. Chondrocytes were transfected with **(a) **a κB reporter or **(b) **a retinoic acid response element (RARE) reporter and treated with or without tumour necrosis factor (TNF)-α (30 ng/ml) and/or all-trans retinoic acid (atRA; 1, 10, or 100 nmol/l) for 24 hours. Panel a: TNF-α-induced nuclear factor-κB (NF-κB) activity was reduced by atRA in a concentration-dependent manner. Panel b: TNF-α had no effect on the concentration-dependent increases in RAR activity. Data are ratios of NF-κB- or retinoic acid receptor (RAR)-regulated firefly luciferase units to constitutive SV40-regulated renilla luciferase units, and are means ± standard error (based on at least three replicates) from a single experiment, representative of three independent experiments. Data were evaluated by one-way analysis of variance and Tukey's multiple comparisons tests. Unlabelled bars or bars labelled with the same lower case letters are not significantly different (*P *> 0.05). RLU, relative luciferase units.

On their own, TNF-α (30 ng/ml) and atRA (10^-9 ^mol/l) reduced Sox-9 activity by about 50%, and together activity was reduced by about 75% (Figure 2a). Because their effects were not completely additive, we determined whether there are interactions between atRA and TNF-α signalling that influence NF-κB or RAR activity. Chondrocytes were transfected with a κB luciferase reporter construct and were treated with TNF-α and/or atRA (Figure 4a). As expected, TNF-α induced NF-κB activity. atRA alone had no effect on basal NF-κB activity. However, atRA significantly inhibited TNF-α-activated NF-κB activity, suggesting that active RARs suppress NF-κB activity.

We next evaluated whether activation of NF-κB influences RAR function. Chondrocytes were transfected with a RARE reporter and treated with TNF-α and/or atRA (Figure 4b). As expected, atRA increased RAR activity in a concentration-dependent manner. TNF-α did not change basal or atRA-induced RAR activity. Thus, under these conditions, NF-κB had no inhibitory effect on RAR activity.

### atRA inhibits binding of DNA by the TNF-α-activated complex

**Figure 5:**
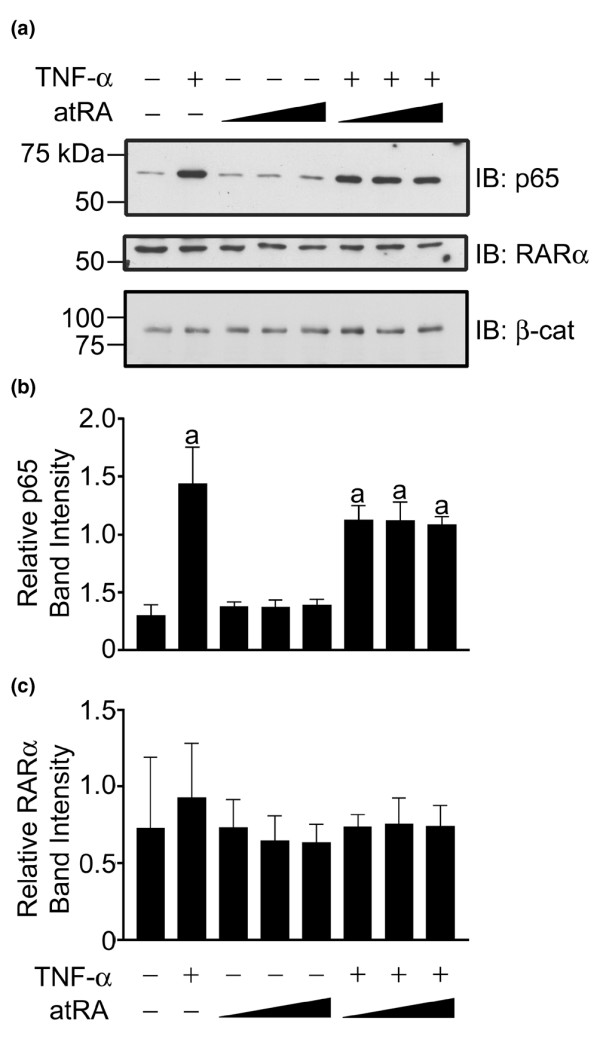
Effects of TNF-α and atRA on nuclear levels of NF-κB and RARα. Chondrocytes were treated with or without tumour necrosis factor (TNF)-α (30 ng/ml) and/or all-trans retinoic acid (atRA; 1, 10, or 100 nmol/l) for 24 hours. **(a) **Nuclear extracts (30 μg) were resolved on a 7.5% polyacrylamide gel and immunoblotted with antibodies against nuclear factor-κB (NF-κB) p65, retinoic acid receptor (RAR)α, or β-catenin (β-cat). Signal intensities for **(b) **NF-κB p65 and **(c) **RARα were quantified by densitometry. Panels **(a) **and **(b)**: TNF-α induced nuclear localization of NF-κB, which was not significantly reduced by atRA. Panels **(a) **and **(c)**: RARα nuclear levels were not significantly altered by treatment with TNF-α or atRA. Panel a: The immunoblots shown are representative of three independent experiments. Panels **(b) **and **(c)**: data are expressed as ratios of intensities of NF-κB or RARα to β-catenin and are means ± standard error. Data were evaluated by one-way analysis of variance and Tukey's multiple comparisons tests. Unlabelled bars or bars labelled with the same lower case letters are not significantly different (*P *> 0.05).

To define further the inhibitory effect of atRA on NF-κB activity, we examined the effects of atRA on nuclear localization of NF-κB and its affinity for DNA. The presence of NF-κB p65 in nuclear extracts from chondrocytes treated with TNF-α and/or atRA was analyzed by immunoblot (Figure 5). TNF-α, but not atRA, induced nuclear localization of the NF-κB p65 isoform (Figure 5a,b). Furthermore, in the presence of TNF-α, treatment of cells with atRA did not significantly reduce the amount of nuclear p65. Moreover, no significant changes in nuclear levels of RARα protein were observed (Figure 5a,c). Thus, reduction in functional activity of NF-κB was not associated with changes in nuclear levels of NF-κB p65 or RARα.

**Figure 6:**
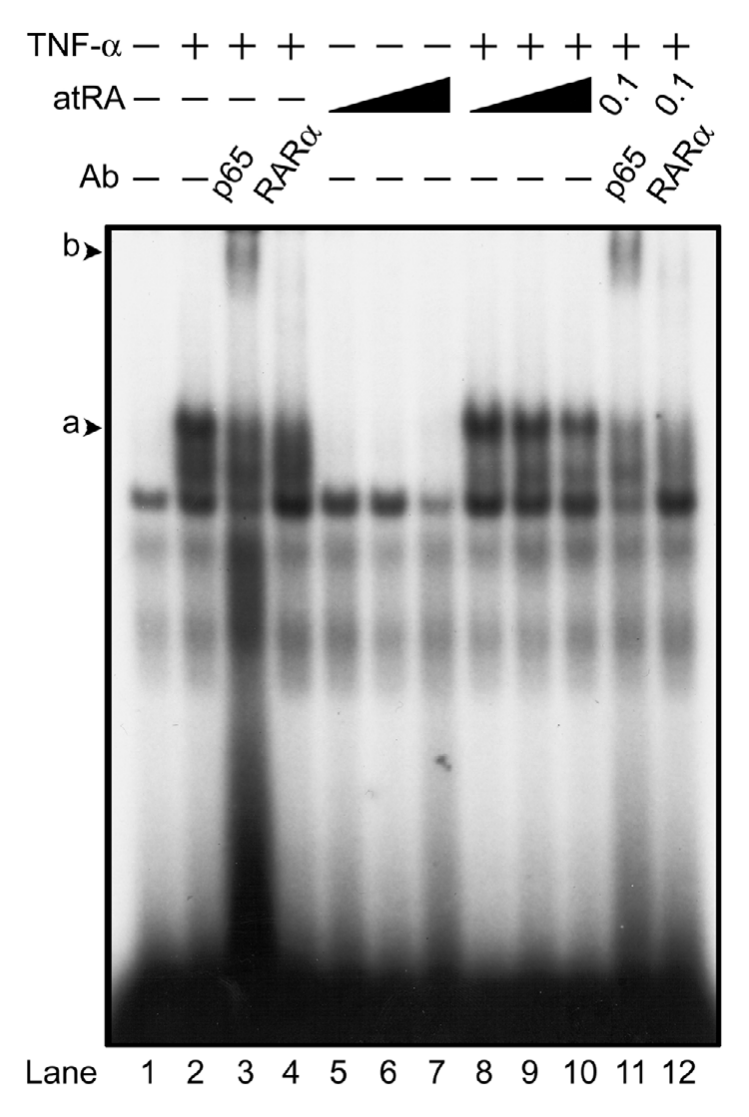
Effect of atRA on NF-κB/DNA binding. Chondrocytes were treated with or without tumour necrosis factor (TNF)-α (30 ng/ml) and/or all-trans retinoic acid (atRA; 1, 10, or 100 nmol/l) for 24 hours. Nuclear extracts were incubated with ^32^P-radiolabelled κB consensus DNA and resolved on a 4% polyacrylamide gel. TNF-α induced the formation of a complex of proteins that contained both nuclear factor-κB (NF-κB) p65 and retinoic acid receptor (RAR)α bound to the κB consensus site (a, lane 2). Addition of antibody against NF-κB p65 gave rise to a supershifted complex (b, lanes 3 and 11). Antibody against RARα interfered with binding of the complex to DNA (lanes 4 and 12). atRA decreased the amount of TNF-α-activated complex bound to the κB consensus site in a concentration-dependent manner (lanes 8 to 10). The TNF-α-activated complex that remained bound in the presence of atRA (0.1 μmol/l) contained p65 and RARα (lanes 11 and 12). Results shown are representative of three independent experiments.

We next evaluated the possibility that RAR activation changes the binding of NF-κB to DNA. Nuclear extracts of cells treated with TNF-α and/or atRA were analyzed by EMSA and supershift/antibody interference assays (Figure 6). TNF-α, but not atRA, induced formation of a complex bound to the κB consensus site that contained the NF-κB p65 isoform. Interestingly, addition of anti-RARα antibody reduced the TNF-α-activated complex, indicating that RARα is a member of this complex (Figure 6; compare lanes 2 and 4, and lanes 10 and 12). Furthermore, atRA decreased the intensity of TNF-α-activated complexes bound to the κB consensus site (Figure 6; compare lanes 2 and 8 to 10). When chondrocytes were treated with both TNF-α and atRA, the complex that remained bound to DNA contained p65 and RARα (Figure 6; compare lanes 10, 11 and 12). Taken together, we conclude that atRA binding to RARα decreases the affinity for DNA of the p65/RARα complex that is formed in response to TNF-α.

### MEKK1 inhibits the effect of atRA on NF-κB functional activity

Changes in transcription factor function can result from alterations in the level or activity of the transcription factor itself or of its required co-factors. Active MEKK1 induces NF-κB nuclear localization by promoting the degradation of IκB [32]. In addition, MEKK1 phosphorylates p300, increasing its histone acetylase activity [33]. Thus, the effect of active MEKK1 on NF-κB and RAR activity was investigated.

**Figure 7:**
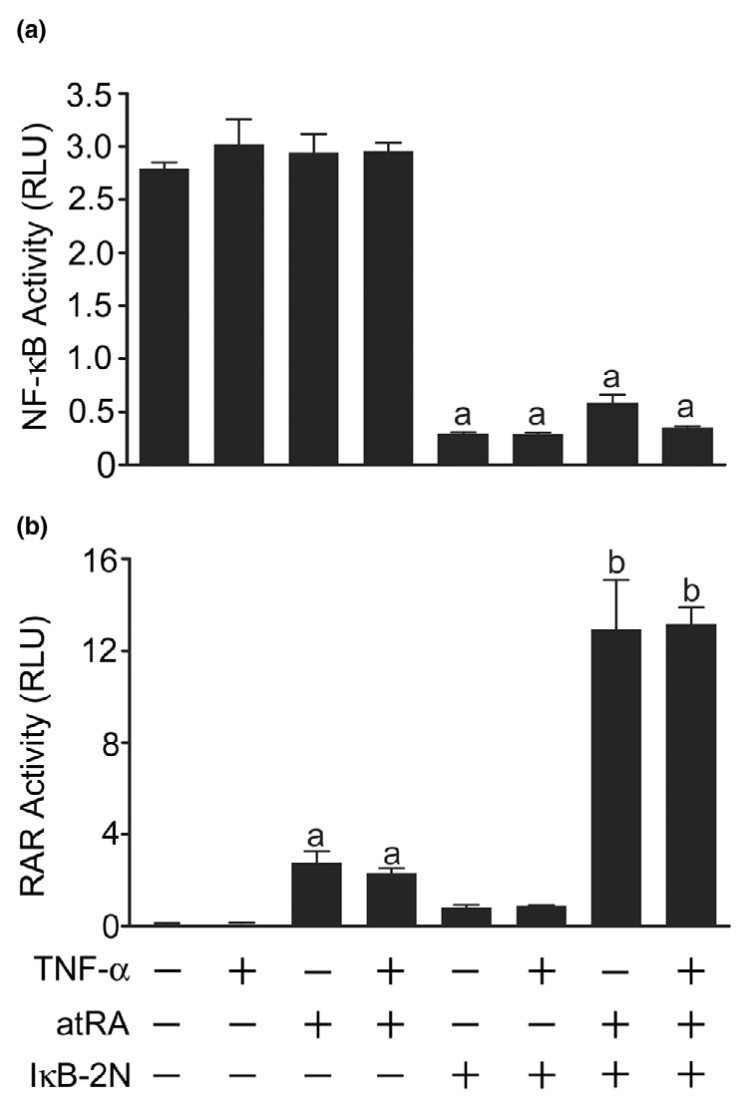
Effect of caMEKK1 on NF-κB and RAR activities. Chondrocytes were co-transfected with a constitutively active mitogen-activated protein kinase kinase kinase (caMEKK)1 expression vector and **(a) **nuclear factor-κB (NF-κB) or **(b) **retinoic acid response element (RARE) reporter vectors. Cells were then treated with or without tumour necrosis factor (TNF)-α (30 ng/ml) and/or all-trans retinoic acid (atRA; 100 nmol/l) for 24 hours. Panel a: caMEKK1 increased basal NF-κB activity and this level was not further increased by TNF-α. atRA had no effect on the level of caMEKK1-induced NF-κB activity. Panel b: atRA significantly increased RAR activity. Co-transfection with the IκB-2N expression vector inhibited NF-κB activity and further increased atRA-induced RAR activity (b, *P *< 0.001). Data are ratios of NF-κB- or RAR-regulated firefly luciferase units to constitutive SV40-regulated renilla luciferase units, and are means ± standard error (*n *= 3) from a single experiment, representative of three independent experiments. Data were evaluated by one-way analysis of variance and Tukey's multiple comparisons tests. Unlabelled bars or bars labelled with the same lower case letters are not significantly different (*P *> 0.05).

Chondrocytes were co-transfected with a caMEKK1 expression construct and either κB or RARE reporter constructs (Figure 7). caMEKK1 dramatically increased basal NF-κB activity. For example, basal NF-κB activity was enhanced approximately 24-fold in the experiment shown (compare first columns in Figure 7a and Figure 4a from the same representative experiment; *P *< 0.001). Treatment of caMEKK1-transfected chondrocytes with TNF-α did not further increase NF-κB activity; however, activity levels were still 4-fold greater than those in cells transfected with reporter alone and treated with TNF-α (compare second columns in Figure 7a and Figure 4a). Interestingly, treatment of caMEKK1-transfected chondrocytes with atRA, alone or in combination with TNF-α, did not reduce NF-κB activity (Figure 7a). Thus, NF-κB function was maximized by caMEKK1 and was also protected from inhibition by atRA.

RAR activity was not altered by expression of caMEKK1 in either the absence or presence of TNF-α (compare first two columns in Figure 7b and Figure 4b). In contrast, atRA-induced RAR activity was increased approximately 11-fold in caMEKK1-transfected chondrocytes compared with cells transfected with reporter alone (compare Figure 7b and Figure 4b; *P *< 0.001). To determine the effect of NF-κB activation by caMEKK1 on RAR function, cells were co-transfected with caMEKK1 and IκB-2N. IκB-2N inhibits NF-κB activation but should not affect other signalling events initiated by caMEKK1. As expected, IκB-2N inhibited NF-κB activity induced by caMEKK1 (Figure 7a). Surprisingly, IκB-2N dramatically increased atRA-induced RAR activity in MEKK1-transfected cells (Figure 7b).

In summary, caMEKK1 increases the functional activity of both NF-κB and atRA-induced RARs. Furthermore, in caMEKK1 expressing cells, atRA does not reduce NF-κB function. Finally, inhibition of NF-κB further enhances the effect of caMEKK1 on atRA-induced RAR function. It is likely that the caMEKK1-induced increases in NF-κB and RAR activity are mediated by hyperactivation of p300.

### caMEKK1 attenuates TNF-α and atRA-induced decrease in Sox9 activity

**Figure 8:**
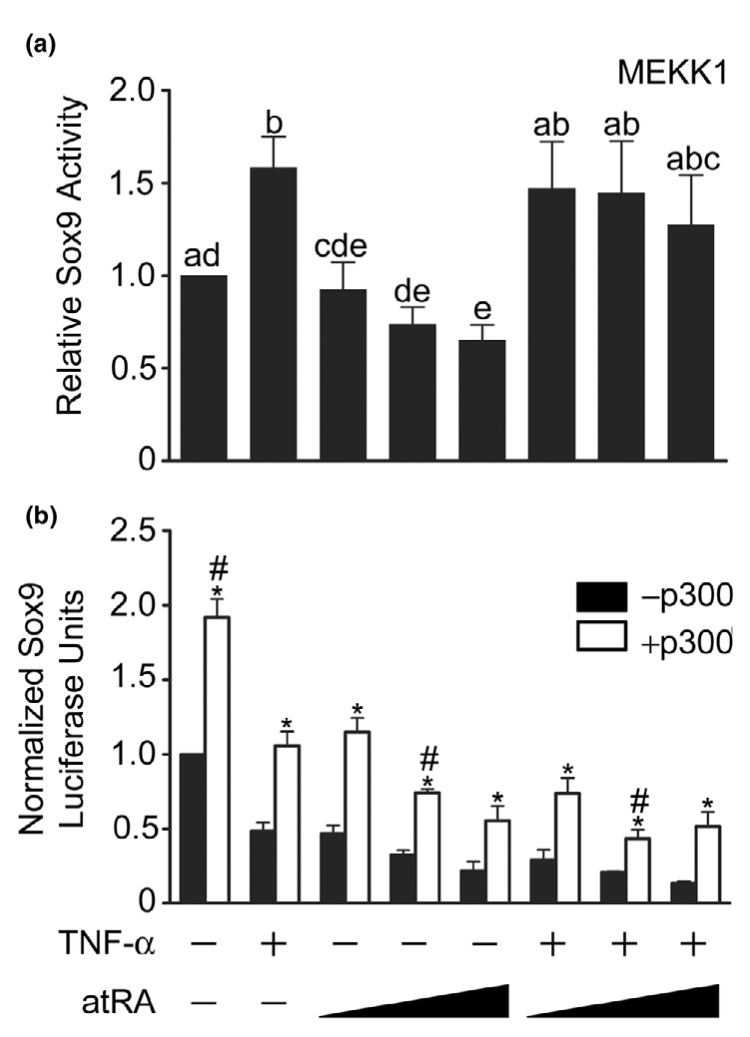
Effect of caMEKK1 and ectopic p300 on Sox9 activity. **(a) **Chondrocytes were co-transfected with the Sox9 reporter and constitutively active mitogen-activated protein kinase kinase kinase (caMEKK)1 expression vector and treated for 24 hours with or without tumour necrosis factor (TNF)-α (30 ng/ml) and/or all-trans retinoic acid (atRA; 1, 10, or 100 nmol/l). TNF-α significantly increased Sox9 activity, whereas the highest concentration of atRA decreased Sox9 activity. Co-treatment with atRA and TNF-α resulted in Sox9 activity levels equivalent to that observed in untreated cultures containing caMEKK1 (first bar). Data are ratios of Sox9-regulated firefly luciferase units to renilla luciferase units normalized as a fraction of the ratio in untreated cultures (first bar), and are expressed as means ± standard error. Data were evaluated by repeated measures analysis of variance and Tukey's multiple comparisons test (three independent experiments). Bars labelled with the same lower case letters are not significantly different (*P *> 0.05). **(b) **Chondrocytes were transfected with Sox9 reporter alone (closed bars) or in combination with p300 expression vector (open bars) and then treated with TNF-α and/or atRA, as indicated. Co-transfection with the p300 expression vector significantly increased Sox9 activity in comparison with similarly treated normal cells (* significant effect of p300, *P *< 0.05). Under most conditions, over-expression of p300 also maintained Sox9 activity at a level comparable to that observed in normal, untreated chondrocytes (first closed bar). Data are Sox9-regulated luciferase units normalized to level in normal, untreated chondrocytes. ^#^Significant difference compared with the first closed bar (*P *< 0.05). Data are means ± standard error (three independent experiments). Data were analyzed by paired *t*-tests.

To investigate the effect of caMEKK1 expression on Sox9 functional activity, cells were co-transfected with the caMEKK1 expression construct and the Sox9 reporter. Over-expression of caMEKK1 significantly increased basal Sox9 activity compared with cells lacking caMEKK1. A comparison of the non-normalized data from the first columns of Figure 8a and Figure 2a revealed that caMEKK1 increased Sox9 activity by 3.7 ± 0.7 fold (mean ± standard deviation; *P *< 0.01; data are from the same series of experiments). Moreover, in contrast to the reductions in Sox9 activity observed in cells lacking caMEKK1 (Figure 2a), TNF-α significantly increased Sox9 activity (Figure 8a). atRA significantly reduced Sox9 activity in cells expressing caMEKK1, but only at the highest concentration used (Figure 8a). In summary, caMEKK1 increased Sox9 functional activity, as it did NF-κB and RAR functional activity. caMEKK1 also reversed the inhibitory effect of TNF-α on Sox9 activity. Finally, the sensitivity of Sox9 to atRA was reduced by caMEKK1 expression. Thus, the activity state of p300, the common co-factor and MEKK1 target, probably plays a vital role in regulating Sox9, NF-κB and RAR function.

### Sox9 functional activity is dependent on availability of p300

We next investigated directly whether availability of p300 contributes to the reduction in Sox9 activity induced by activation of NF-κB and RARs. Cells were co-transfected with the Sox9 reporter and a p300 expression construct. Cells over-expressing p300 exhibited significantly increased Sox9 activity compared with cells transfected with Sox9 reporter alone (Figure 8b). Ectopic p300 expression did not prevent reductions in Sox9 activity in response to TNF-α and atRA. However, under most conditions, over-expression of p300 maintained Sox9 activity at a level comparable to that observed in normal, untreated chondrocytes. Thus, increasing the availability of p300 increases Sox9 activity even when NF-κB and RARs are active.

## Discussion

### Regulation of Sox9 activity through activation of NF-κB and RARs

Sox9 is required for expression of cartilage matrix genes [4-6]. We demonstrated a reduction in Sox9 activity after treatment with atRA or TNF-α, consistent with previous reports of the individual effects of these factors in condensing mesenchymal cells and articular chondrocytes [13,28]. We extended these studies by treating chondrocytes with both atRA and TNF-α, revealing a further decrease in Sox9 activity compared with each factor alone. Such a loss could be attributable to either a reduction in Sox9 protein level or altered activity. However, we found no effect of TNF-α or atRA on Sox9 nuclear protein levels. Moreover, TNF-α did not alter the level of protein complexes bound to the *Col2a1 *48-bp minimal enhancer sequence. Consistent with these findings, previous studies have shown that levels and stability of Sox9 mRNA are not altered over a 24-hour period following treatment of mouse chondrocytes with 0.1 μmol/l atRA [34], which is the highest concentration used in the present study. In other studies, TNF-α partially reduces Sox9 protein levels over a period of 8 hours through a NF-κB-dependent, post-transcriptional mechanism in mouse chondrocytes [15,35]. In our system, changes in activity of Sox9 in response to TNF-α and atRA are probably due to alternate modes of regulation, independent of changes in protein levels or DNA binding.

For full gene transactivation function, Sox9 requires the recruitment of the histone acetylase p300 [25,36]. p300 is a common co-factor that is required by multiple transcription factors, including Sox9, NF-κB and RARs, for full activity. In the present study, ectopic expression of p300 increased basal Sox9 activity. Moreover, increasing p300 levels maintained Sox9 activity in the presence of TNF-α and atRA, at a level comparable to that observed in normal, untreated cells. The over-expression of p300 did not attenuate the reductions in Sox9 activity induced by NF-κB and RAR activation, suggesting that greater expression of p300 is necessary to overcome these reductions. In contrast, caMEKK1, which hyperactivates p300, attenuated the reductions in Sox9 activity; this suggests that both p300 levels and activity are limiting in chondrocytes.

Further evidence that p300 is a limiting co-factor in chondrocytes comes from studies of genetically modified mice. Animals heterozygous for *p300 *or its closely related family member *Cbp *exhibit growth abnormalities, including defects in bone and cartilage [37,38]. Consequently, activation of transcription factors that sequester p300 (such as NF-κB and RARs) may suppress cartilage matrix synthesis.

Reductions in p300 availability or activity may contribute to the teratogenic effects of retinoids. Imbalances in atRA have major effects on chondrocyte development. atRA inhibits the expression of Sox9 in chondroprogenitor cells, resulting in major cartilage and bone abnormalities [39]. In addition, acute arthritic symptoms can arise when patients are treated with retinoids for dermatologic disorders such as acne [40,41], which is consistent with the effects of retinoids in mice [19] and is possibly due to reduced cartilage matrix production [18].

### Interactions between NF-κB and RARs

In the present study we observed decreases in the functional activity of NF-κB in the presence of atRA, which is consistent with the decreases in NF-κB/DNA interactions seen in macrophages treated with the atRA analogue TTNPB. In addition, over-expression of p300 rescues NF-κB activity reduced by 9-cis RA activation of retinoid X receptors [22]. Our results suggest that atRA-activated RARs not only limit p300 availability for Sox9 but also limit its availability for activated NF-κB. On the other hand, NF-κB activation can influence retinoid responsive elements. For example, over-expression of NF-κB p50 or p65 decreases 9-cis RA-activated retinoid X receptor activity in macrophages [22]. However, we found no reciprocal reduction in RAR activity when chondrocytes were treated with both TNF-α and atRA.

### Regulation of transcription factor activity by p300

Differences in the affinity for p300 may contribute to the pattern of regulation of transcription factor activity observed in the present study. Because atRA reduced both Sox9 and NF-κB activity in chondrocytes, active RARs may have a higher affinity for p300 compared with Sox9 or NF-κB. Thus, active RARs would be able to transactivate genes optimally, even when NF-κB has translocated to the nucleus. NF-κB and Sox9 appear to have similar command of p300, because Sox9 activity was reduced by approximately 50% when NF-κB was activated. However, the demand for p300 is controlled not only by relative affinity but also by relative stoichiometry of the transcription factors activated in the nucleus.

Manipulating the acetylase activity of p300 can reveal its role in regulating the function of transcription factors. In the present study, caMEKK1 enhanced the functional activities of NF-κB, RARs and Sox9. One explanation for these increases is post-translational modification of p300. p300 is phosphorylated in both the carboxyl-terminal and amino-terminal regions by a kinase in the MEKK1 pathway, resulting in increased acetylase activity [33]. In the present study, caMEKK1 increased NF-κB, atRA-induced RAR and Sox9 activity, suggesting that hyperactive p300 can circumvent its own limiting levels.

Another explanation for increased transcription factor activity in response to active MEKK1 is increased nuclear translocation of specific transcription factors. Active MEKK1 initiates the degradation of IκB, freeing NF-κB to enter the nucleus [32,42]. Therefore, the maximization of NF-κB activity induced by caMEKK1 observed in the present study may result from activating more NF-κB than is possible with TNF-α alone. Under basal conditions, NF-κB activation did not reduce RAR activity; however, when NF-κB was maximally activated by caMEKK1, it did suppress RAR activity. This effect is likely due to increased nuclear NF-κB sequestering a greater proportion of p300.

The additional increases to Sox9 activity induced by caMEKK1 and TNF-α observed in the present study suggest other post-translational modifications to Sox9 or p300 by signals initiated by TNF-α that are independent of hyperactivated p300. The possible mechanisms associated with this regulation require further investigation.

Increases in transcription factor activity can also result from direct acetylation by p300, as seen with NF-κB [43]. Human NF-κB p65 has three lysine (K) residues that are targets of acetylation by p300, each serving a different role. Acetylation of K221 is required for DNA binding and, in conjunction with acetylation of K218, inhibits association with IκBα. Reduced p300 availability or activity would decrease acetylation at the K221 site, thereby suppressing NF-κB binding to DNA. This mechanism could contribute to the reduction in binding to DNA of the TNF-α-activated complex observed under conditions in which chondrocytes were subjected to both TNF-α and atRA. Acetylation at K218 is probably unaffected, because we did not observe any changes in nuclear localization of NF-κB p65 in the presence of both TNF-α and atRA. More interestingly, acetylation of K310 is required for full NF-κB transcriptional activity. Hyperactivation of p300 induced by caMEKK1 could cause changes in NF-κB acetylation, leading to maximal NF-κB functional activity. In particular, increased acetylation of K310 may explain part of the increase in NF-κB activity that we observed when caMEKK1 is expressed in the chondrocytes. Thus, active MEKK1 may increase the acetylase function of p300, allowing associated transcription factors (such as those studied here) to overcome the limiting levels of p300 and increase their activity.

## Conclusion

In this study, we investigated how active transcription factors, sharing common co-factors, regulate gene expression. We have shown that Sox9 activity and expression of cartilage matrix genes are limited by coincident activation of NF-κB and RARs. We have also shown that Sox9 activity can be maintained in the presence of TNF-α or atRA by ectopically over-expressing p300 or increasing p300 acetylase function. Thus, crosstalk among Sox9, NF-κB and RARs involving p300 represents a key mechanism for the coordinated regulation of matrix gene expression in chondrocytes. Moreover, p300 may be a relevant target for therapeutic intervention in arthritis.

## Abbreviations

atRA = all-trans retinoic acid; bp = base pair; caMEKK = constitutively active mitogen-activated protein kinase kinase kinase; EMSA = electrophoretic mobility shift assay; IκB = inhibitor of nuclear factor-κB; NF-κB = nuclear factor-κB; PCR = polymerase chain reaction; qPCR = quantitative real-time polymerase chain reaction; RAR = retinoic acid receptor; RARE = retinoic acid response element; Sox = Sry-type high mobility group box; TNF = tumour necrosis factor.

## Competing interests

The authors declare that they have no competing interests.

## Authors' contributions

JSR carried out transfection studies, EMSAs and immunoblots, and performed all statistical analyses and drafted the manuscript. JCK participated in some of the initial transfection studies. AJG participated in investigating Sox9 through EMSA. SMB conceived of the study, participated in its design and coordination, and helped to draft the manuscript. JSR, JCK and AJG read and approved the final manuscript.
